# With Appreciation

**DOI:** 10.1002/jcsm.12519

**Published:** 2019-12-10

We thank the following individuals who served as manuscript reviewers for The Journal of Cachexia, Sarcopenia and Muscle (JCSM) in 2019.

We highly appreciate their efforts. Fair, conscientious, and timely peer reviews contribute to the success of JCSM.

The Editors.

### 6 and more reviews

Paola Costelli

Boel De Paepe

Aminah Jatoi

Takao Kato

Fabio Penna

Kunihiro Sakuma

### 3‐5 reviews

Pasquale Abete

Volker Adams

Odessa Addison

Stephen Alway

Hidenori Arai

Atsushi Asakura

Hideo Baba

Vickie Baracos

Thiago Barbosa‐Silva

Daniel Bechet

Rosanne Beijers

Mauricio Berriel Diaz

Richard Bohannon

Andrea Bonetto

Federico Bozzetti

Justin Brown

Olivier Bruyere

Eduardo Cadore

James Carson

Lydie Combaret

Srinivasan Dasarathy

Jackie Dawson

Hans Degens

Marcelo Dos Santos

H. Alexander Ebhardt

Dirk Fischer

James Fluckey

Giacomo Garibotto

Marcus Goncalves

Nicholas Greene

Denis Guttridge

Shinji Hatakeyama

Michiyoshi Hatanaka

Troy Hornberger

Satoshi Ikeda

Carsten Jacobi

Andrew R. Judge

Boris Jung

Anu Kauppinen

Seyyed M. Reza Kazemi‐Bajestani

Paul Kemp

Andy Khamoui

Scot Kimball

Nicole Kiss

Siegfried Labeit

Fulvio Lauretani

Alessandro Laviano

Lorenz Lehmann

Lisa Martin

Anna Maria Martone

Anselmo Moriscot

John Morley

Yoshinaga Okugawa

Anna Pakula

Evasio Pasini

Goncalo Pereira

Rosanna Piccirillo

Sandra Schütze

Marília Seelaender

Rajan Singh

Richard Skipworth

Andrew Stewart Coats

Claudia Suemoto

Bella Talwar

Klaske van Norren

Jeroen van Vugt

Stefano Volpato

Xiaona Wang

Teresa Zimmers

Jana Zschüntzsch

### 1‐2 reviews

Eirik Aahlin

Eeva Aartolahti

Santanasto Adam

Christopher Adams

Yoshihiro Akashi

Ziyad Al‐Aly

Matthew Alexander

Poorna Anandavadivelan

Lars Andersen

Ivan Aprahamian

Robert Arpke

Keith Avin

Umesh Badrising

Esther Barreiro

Maximilien Barret

Philipe Barreto

Jeannette Beasley

Charlotte Beaudart

Jose Berciano

David Berger

Henri Bernardi

Sue Bodine

Laura N. Borodinsky

Olivier Bouché

Thierry Boulain

T. Scott Bowen

Scott Brakenridge

Jeffrey Brault

Ellen Breen

Thomas Brioche

Marco Brotto

Silvia Brunelli

Olivier Bruyère

Martin Burtscher

Philip Calder

Daniel Cannon

John Carbone

Pierre Carlier

Robson Carvalho

Mariana Cavagnari

Peggy Cawthon

Lucely Cetina

Victor Chang

Antonio Cherubini

Stephanie Chevalier

Jesper Frank Christensen

Paul Coen

Peter Collins

Marcia Cominetti

Maria Isabel Correia

Alfonso Cruz‐Jentoft

Dirk De Ruysscher

Marian de van der Schueren

Gilles Defraene

Patrice Delafontaine

Elsa Dent

Nicolaas Deutz

Mauro Di Bari

Francesco Dioguardi

Marlou Dirks

Wolfram Doehner

Jason Doles

Sami Dridi

Wilfred Druml

Hervé Dubouchaud

Georg Duda

Debapriya Dutta

Carrie Earthman

Nicole Ebner

Ahmed Elhakeem

Marielle Engelen

Hirayuki Enomoto

Karyn Esser

William Evans

Nir Eynon

Richard Ferguson

Alessandra Ferlini

Elisabetta Ferraro

Julio Cesar Ferreira

Li Filippin

Bertrand Fougère

Gabriel Francisco Pereira Aleixo

Frits Franssen

Paula Freire

Alberto Frisoli

Matheus Garcia

Jose Garcia

Ghislaine Gayan‐Ramirez

Jürg Gertsch

Hugo Giambini

Francois Goldwasser

Maria Cristina Gomes‐Marcondes

Maria Cristina Gonzalez

Craig Goodman

Loncar Goran

Brad Gordon

Nandu Goswani

Gilles Gouspillou

Alexander Grimm

Bruno Gualano

Jenny Gunton

Cheng Guo

Anne Güttsches

Dirk Habedank

David Hammers

Der‐Sheng Han

Luciana Hannibal

Mette Hansen

Justin Hardee

Jörg Heineke

Russell Hepple

Steven Heymsfield

Daniel Hirai

Sandra Holasek

Kieren Hollingsworth

Corinne Horlings

Zhaoyong Hu

Harinder Hundal

Sabah Hussain

Chikwendu Ibebunjo

Hikaru Ihira

Katsuya Iijima

Mikel Izquierdo

Lee Jones

Anne Jouinot

Paweł Kabata

Toshimi Kaido

Kam Kalantar

Nuray Kanbur

Julie Ann Kemp

Eva Kiesswetter

Hiroshi Kimura

Serkan Kir

Brandon M. Kistler

Amanda Kleiman

Yann Klimentidis

Gangjee Ko

Jan Koch

Masaaki Konishi

Laetitia Koppe

Kazuo Koyanagi

Morten Tange Kristensen

Ashok Kumar

Mitja Lainscak

Barry Laird

Ramon C. J. Langen

Anis Larbi

Wei Ling Lau

Michaël Laurent

Carl Lavie

Jae‐myeong Lee

Helmar Lehmann

Darryl Leong

William Leslie

Stef Levolger

Yi‐Ping Li

Adam Lightfoot

Jeremy Loenneke

Goran Loncar

Audrey Loumaye

Markus Luedi

Vladimir Lyadov

Matthew Maddocks

Stefania Maggi

Robert Mak

Theodore Malmstrom

Giovanni Mantovani

Daniela Mari

Daniel Marks

Nicolas Martinez‐Velilla

Yorgi Mavros

Anne May

John McCarthy

Donald McMillan

Christa Meisinger

Kim Meredith

Jeffrey Metter

Thomas Meyer

William E. Mitch

Junaith Mohamed

Fiammetta Monacelli

Aldo Montano‐Loza

Beatrice Morio

Jasper Morrow

Remi Mounier

Marina Mourtzakis

Karlien Mul

Kevin Murach

Kate Murphy

Maurizio Muscaritoli

David Mutch

Yoko Narasaki

Sara Nejatinamini

Jakob Nielsen

Bernd Niemann

Sagar U. Nigwekar

Enzo Nisoli

Kristina Norman

Piotr Nowaczyk

Thomas OConnell

Kook‐Hwan Oh

Kay Ohlendieck

Darlington Okonko

Paulo J. Oliveira

Camila Oliveira

Ismail Ozsoy

Lynn Panton

Carolyn J Peddle‐McIntyre

Suzette Pereira

Dominik Pesta

Susanne Petri

Michele Petruzzelli

Leonidas Phylactou

Karteek Popuri

Scott Powers

Tomasz Powrózek

Carla Prado

Olga Prokopchuk

Melissa Puppa

Sarah Purcell

Enkhsaikhan Purevjav

Robinson Ramirez‐Vélez

Blake Rasmussen

Paula Ravasco

Sander Rensen

Connie Rhee

Sandra Maria Ribeiro

Erik Richter

Thomas Riediger

Christian Riehle

Fernando Romeiro

Hamilton Roschel

Bob Roshanravan

Urs Ruegg

Markus Ruegg

Dolores Sanchez‐Rodriguez

Marcus Sandri

Nadja Scherbakov

Jens Schmidt

Brad Schoenfeld

Annemie Schols

Michael Schwarzer

Stylianos Scordilis

Adam Sharples

Ken Shirabe

Ng Shyh‐Chang

Mario Siervo

Elina Sillanpaa

Adam Sima

Parco Siu

Tora Solheim

Pietro Spitali

Jochen Springer

Ivan Stankovic

Michael Steiner

Georg Stettner

Katharina Stoeck

Elani Streja

Sara Tagliaferri

Daniel Taillandier

Yohei Takai

Tetsuro Tamaki

Puneeta Tandon

Anne‐Julie Tessier

Jean‐Paul Thissen

Jeanette Thom

David Thomas

Erkan Topkan

Miguel Toscano Rico

Susan Treves

Dominik Uehlinger

Gregorio Valdez

Vanessa Valdiglesiass

Peter van der Meer

David van Dijk

Ardy van Helvoort

Luc van Loon

Bolormaa Vandanmagsar

Hanna Vankova

Aphrodite Vasilaki

Vasilis Vasiliou

Nick Vaziri

Frank Verahaegen

Lex Verdijk

Davide Liborio Vetrano

José M. Villalba

Jose Vina

Kristian Vissing

Konstantinos Volaklis

Christine von Arnim

Hidetaka Wakabayashi

Noah Weisleder

Steven Welc

Yefei Wen

Barbara Wessner

Michael Wiggs

Saffron Willis‐Owen

Kenneth Wilund

Miles Witham

Christian Witt

Klaus Witte

Felix Woitek

Robert Wolfe

Kai Wollert

Jean Woo

Chih‐Hsing Wu

Rob Wust

Qian‐Li Xue

Sumio Yamada

Mee‐Sup Yoon

Tatsuki Yoshimatsu

Zhen Yu

Yao Zhu

Cheng‐le Zhuang

Carmine Zoccali

